# GLIMSI: A real-world, multicenter study assessing the effectiveness and safety of Sitagliptin + Glimepiride + Metformin FDC in Indian patients with Type 2 diabetes

**DOI:** 10.1371/journal.pone.0337107

**Published:** 2026-02-06

**Authors:** Kunal Zaveri, Girish Kulkarni, Rathish Nair, Govarthanan Shanmugan, Shubhada Amol Dharmadhikari, R. P. S. Makkar, Rahul Jalgaonkar, B. D. Chatterjee, Krishnaprasad Korukonda, GLIMSI Investigator(s) Study Panel

**Affiliations:** 1 Internal Medicine, Vishesh Clinic, Ahmedabad, Gujarat, India; 2 Medical Affairs, Torrent Pharmaceuticals Ltd, Ahmedabad, Gujarat, India; 3 Medical Affairs, Torrent Pharmaceuticals Ltd, Ahmedabad, Gujarat, India; 4 Endocrinology, Dr. Govarthanan’s Diabetic Clinic, Chennai, Tamil Nadu, India; 5 Endocrinology, Shivalay Diabetic clinic, Kolhapur, Maharastra, India; 6 Internal Medicine, Life Line Hospital, East Delhi, New Delhi, India; 7 Endocrinology, Health Insight, Dombivli, Maharashtra, India; 8 Endocrinology, Sundaram Clinic, Bardhaman, West Bengal, India; 9 Medical Affairs, Torrent Pharmaceuticals Ltd, Ahmedabad, Gujarat, India; The Chinese University of Hong Kong, HONG KONG

## Abstract

**Background:**

Type-2-diabetes-mellitus (T2DM), often linked to obesity, raises risk of microvascular and macrovascular complications. International guidelines recommend triple-therapy to reach haemoglobin A1c (HbA1c) targets when dual therapy fails to adequately control blood glucose levels. Sitagliptin, enhances glycaemic control by prolonging incretin action, boosting insulin secretion, and lowering glucagon levels. When combined with glimepiride and metformin this triple-therapy targets multiple mechanisms. This study evaluated the effectiveness and safety of this combination for improved T2DM management in Indian patients.

**Method:**

This real-world, multicentre, observational chart review evaluated the efficacy and safety of a triple fixed-dose combination therapy in 1235 adult patients with T2DM across 194 clinical sites in India. Data were retrospectively extracted from patient records over a 12-week period. Descriptive and analytical statistics was applied for the study endpoints using SPSS ver. 29.0.1.0(171) and Microsoft Excel 2019.

**Result:**

The study population had a mean age of 56.89 ± 10.29 years, with 64.70% reporting a family history of type 2 diabetes mellitus (T2DM). Smoking was identified as a prominent risk factor, affecting 38.65% of participants. Significant improvements were observed in glycemic parameters over 12 weeks: HbA1c levels decreased from 8.20 ± 0.60% to 7.08 ± 0.77% (p < 0.0001), fasting blood glucose (FBG) from 188.54 ± 47.59 mg/dL to 146.01 ± 41.53 mg/dL (p < 0.0001), and 2-hour postprandial plasma glucose (PPG) from 234.74 ± 50.40 mg/dL to 179.40 ± 42.51 mg/dL (p < 0.0001). Additionally, body weight significantly reduced from 75.99 ± 8.67 kg to 74.76 ± 9.07 kg (p < 0.0001). No significant safety concerns identified during the treatment period.

**Conclusion:**

The triple-combination therapy (sitagliptin, glimepiride, and metformin) demonstrated superior efficacy in achieving glycemic control, as evidenced by significant reductions in HbA1c, fasting blood glucose (FBG), and postprandial plasma glucose (PPG). Furthermore, the therapy facilitated meaningful weight reduction, highlighting its clinical utility as a comprehensive therapeutic option for managing glycemic parameters in both T2DM with overweight and normal-weight patients.

## Introduction

India is currently recognized as the diabetes capital of the world, with approximately 77 million individuals affected by the condition [1]. Globally, 537 million adults are living with diabetes (1 in 10 individuals)[2], and this number is expected to rise to 643 million by 2030 and 783 million by 2045 [3]. Type 2 diabetes mellitus (T2DM) is closely linked to microvascular complications such as retinopathy, nephropathy, and neuropathy and macrovascular complications, including ischemic heart disease, peripheral vascular disease, and cerebrovascular disease. These complications lead to organ and tissue damage in about one-third to one-half of diabetic individuals [4,5].

Insulin resistance and impaired insulin secretion remain the main defects in T2DM, along with six other pathophysiological abnormalities, termed as “ominous octet”, which contribute to the dysregulation of glucose metabolism. The various pathogenetic disturbances in T2DM necessitate the use of multiple antidiabetic agents in combination to maintain normoglycemia [6,7]. American Diabetes Association (ADA) and the Research Society for the Study of Diabetes in India (RSSDI) guidelines recommend early combination therapy for T2DM patients who fail to achieve glycemic targets with monotherapy, such as metformin, especially in cases where the disease is progressive [8,9]. Studies have demonstrated that patients on triple therapy exhibit significantly improved glycaemic parameters, including HbA1c and fasting blood glucose (FBG), compared to those on dual therapy, making this approach an effective strategy for comprehensive management [10]. Globally recognized guidelines, including the ADA and the European Association for the Study of Diabetes (EASD), recommend incorporating a third agent to reach hemoglobin A1c (HbA1c) targets when dual therapy fails to control blood glucose levels adequately [11].

Achieving and maintaining good glycemic control is crucial to preventing or delaying the onset of diabetes-related complications. Poor glycemic control in patients with T2DM has been strongly linked to an increased risk of both microvascular and macrovascular complications. A key therapeutic objective in management T2DM is to optimize glycemic control and minimize the risk of long-term complications. Findings from the LANDMARC study indicate that biguanides and sulfonylureas remain the most frequently prescribed oral antidiabetic drugs (OADs). In this context, the use of insulin-sparing agents such as dipeptidyl peptidase-4 (DPP-4) inhibitors may serve as appropriate add-on therapies [12]. Despite global and national guideline endorsements for early combination therapy, especially triple-drug regimens, the availability of robust randomized controlled trials (RCTs) and real-world evidence (RWE) evaluating the fixed-dose combination (FDC) of sitagliptin, glimepiride, and metformin remains limited. Observational surveys and small-scale studies in Indian settings suggest improved glycemic outcomes and adherence with triple therapy [13–16]. However, comprehensive RCTs and large-scale RWE studies assessing the safety, efficacy, and comparative effectiveness of this specific FDC in diverse Indian populations are lacking, highlighting the need for well-designed, large-scale studies to validate the clinical utility of triple FDCs in routine diabetes care.

This study aims to evaluate the real-world efficacy and safety of a fixed-dose combination (FDC) of sitagliptin, glimepiride, and metformin for achieving optimal glycemic control in patients with T2DM who have HbA1c levels between 7% and 9%. This includes both treatment-naïve patients and those who are uncontrolled on dual therapy.

## Methods

### Ethical consideration

This multicentre, retrospective, observational chart review was conducted in compliance with international and national regulatory requirements, including the Declaration of Helsinki (2013), Good Clinical Practice (GCP) guidelines, and the International Conference on Harmonisation (ICH) E6(R2) guideline. The study also adhered to the Indian regulatory framework, including the New Drugs and Clinical Trials Rules (2019) and the Indian Council of Medical Research’s (ICMR) national ethical guidelines for biomedical and health research involving human participants (2017). Prior to study initiation, ethics committee approval was obtained from the Sangini Hospital Ethics Committee (EC Registration number: ECR/147/Inst/GJ/2013/RR-24). The study was registered with the Clinical Trials Registry of India (CTRI/2024/03/064004), ensuring transparency and accountability. The data collection forms (DCFs) were deidentified and anonymized at the site level by the respective site Co-Investigators before being shared with the research team. No author had access to identifiable participant information at any point during or after data collection.

### Study design and population

**Fig 1:**
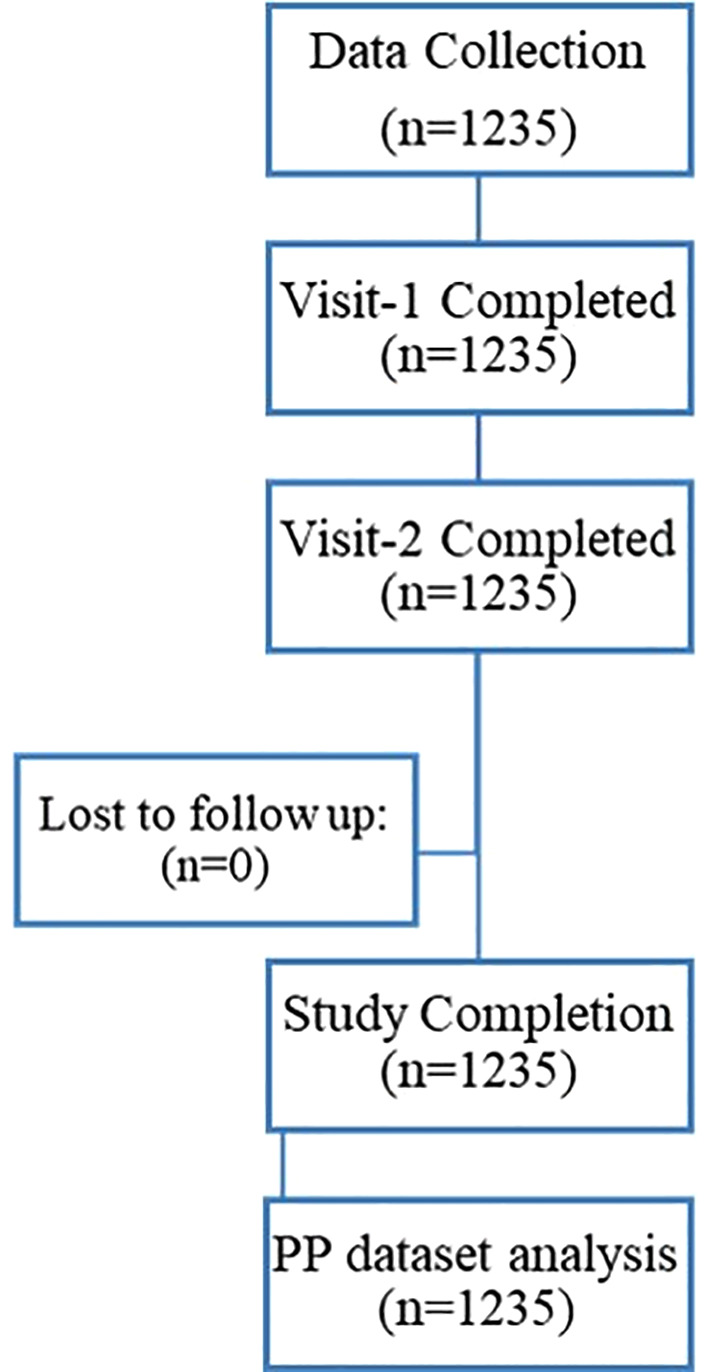
Patients disposition chart. A total of 1235 patients with type 2 diabetes mellitus (T2DM) were included at baseline. All patients completed Visit 1 and Visit 2 assessments, with no loss to follow-up. Consequently, 1235 patients completed the study and were included in the per-protocol (PP) dataset analysis.

This multicentre, retrospective, observational chart review was a post-marketing, real-world evaluation of the safety and effectiveness of a fixed dose combination (FDC) therapy in patients with T2DM. A total of 1235 patients with T2DM were enrolled from 194 sites across India, providing a diverse and representative patient population (Fig 1).

The study population consisted of male and female patients aged 18–85 years with T2DM. Eligible patients included those with inadequate glycemic control on dual therapy (Glimepiride + Metformin or Sitagliptin + Metformin) with HbA1c levels between 7.5% and 8.5%, as well as treatment-naive patients with HbA1c levels ranging from 7.0% to 9.0%. Patients with elevated FBG levels (>126 mg/dL) and postprandial glucose (PPG) levels (>200 mg/dL) were also included. The exclusion criteria for the study included patients with a history of Type 1 diabetes, those who had received insulin treatment within 8 weeks prior to screening and individuals with renal dysfunction (creatinine clearance <60 mL/min) or proteinuria. Additionally, patients with a history of hypersensitivity or intolerance to Sitagliptin, Glimepiride or Metformin, as well as those who were pregnant or lactating were excluded. Patients with congestive heart failure, unstable angina or acute coronary syndrome within the past 6 months were also excluded from the study.

The primary objectives of the study were to evaluate the mean change in HbA1c from baseline to 12 ± 1 weeks of treatment. Additionally, the mean changes in FBG and 1-hour and 2-hour PPG levels were assessed from baseline to 4 ± 1 week and 12 ± 1 weeks of follow-up. The mean change in body weight after 12 ± 1 weeks of treatment was also measured from baseline. The secondary objectives included assessing treatment adherence and compliance. A safety assessment was conducted to evaluate the tolerability of the treatment. Additionally, the Physician Global Assessment (PGA) was used to measure improvement in patient condition, along with an evaluation of user experience throughout the study period.

### Study procedures

Retrospective data were extracted from patient medical records for the period between 25 January 2024 and 25 April 2024. Eligible patients with T2DM who had been prescribed a fixed dose combination of sitagliptin, glimepiride and metformin were identified across participating sites. Baseline information included demographic details, medical history, prior treatment and laboratory parameters such as fasting blood glucose (FBG), postprandial glucose (PPG), glycated hemoglobin (HbA1c) and body weight. Clinical records documenting routine follow-up visits at 4 weeks and 12 weeks after initiation of triple therapy were reviewed. At the 4-week and 12 weeks of follow-up, data on FBG, PPG, body weight and recorded safety parameters were extracted. Additionally, HbA1c and Physician Global Assessment (PGA) scores were extracted on 12 weeks. Treatment adherence had been monitored through entries in the case record forms (CRFs). Patients who had completed the prescribed treatment period and had documented attendance for all scheduled follow-up visits were included in the analysis. The study protocol and case report form were shared with the study site before initiation to maintain the standardization. Data were manually transcribed into standardized, ethics-approved CRFs by site investigators and subsequently reviewed for completeness and accuracy. All blood biochemistry analysis were conducted at NABL-accredited laboratories to ensure quality and reliability of results. Adverse events were qualitatively abstracted from available medical records documented by treating physicians.

### Intervention

The study medication consisted of a fixed dose combination of sitagliptin, glimepiride and metformin tablets (Torrent Pharmaceuticals Limited, India), administered orally once daily.

### Statistical analysis

All statistical analyses were performed using Microsoft Excel and SPSS version 29.0.1.0 (IBM, USA). Continuous variables, such as age, weight and height were analysed using descriptive statistics and presented as mean ± standard deviation (SD). Symptom score improvements were summarized as median values. Categorical variables, including baseline characteristics, prescription analysis and drug usage patterns were reported as frequencies (n) and percentages (%). Endpoints, such as glycemic parameters (HbA1c, PPG, FBG) and body weight were treated as continuous variables. The significance of changes in these parameters was assessed using Student’s paired t-test with a two-tailed p-value <0.05 considered statistically significant. Subgroup analyses were conducted in a post hoc and exploratory manner based on available data.

In addition, a multivariate multiple regression analysis was performed to examine the association between baseline clinical characteristics (e.g., demographic and metabolic parameters) and multiple dependent glycemic outcomes, including changes in HbA1c, FBG and PPG. This method accounted for the intercorrelation among dependent variables and enabled simultaneous assessment of predictors influencing the overall glycemic response to treatment.

## Results

### Baseline patient characteristics and prescription patterns

This real-world assessment enrolled 1235 patients with T2DM, comprising 955 (77.33%) males and 280 (22.67%) females. The mean age of the study population was 56.93 ± 10.28 years with a mean weight and height of 76.00 ± 8.66 kg and 165.29 ± 7.67 cm respectively. The majority of patients (43.64%) had a T2DM duration of 1–3 years and smoking (38.46%) was the most common identified risk factor. Chronic kidney disease (CKD) (25.51%) was the most prevalent comorbid condition. Baseline characteristics and patient demographics are presented in Table 1. In terms of treatment patterns, the majority of patients (60.32%) received a morning dose of the sitagliptin + glimepiride + metformin combination. Telmisartan (64.13%) was the most commonly prescribed angiotensin II receptor blocker (ARB), while rosuvastatin (23.08%) and atorvastatin (20.16%) were the most frequently prescribed statins. Further details on prescription patterns and treatment utilization are presented in S1 and S2 Tables in S1 File.

**Table 1:** Patient demographics and baseline characteristics. Patient history including demographics, family history, disease duration, identifiable risk factors, comorbid conditions, and complications.

Patient history		Percentage
Variable		N = 1235
**Demographics**	**Age (years) (mean, SD)**	56.93 (10.28)
	**Height (cm) (mean, SD)**	165.29 (7.67)
	**Weight (kg) (mean, SD)**	76.00 (8.66)
	**BMI (kg)/m** ^**2**^ **(mean, SD)**	27.86 (3.74)
	**Gender (n, %)**	Male: 955 (77.33%)
		Female: 280 (22.67%)
**Family history of Diabetes**	**No**	65.10%
	**Yes**	34.90%
**Duration of Diabetes**	**< 1 years**	15.55%
	**1-3 years**	43.64%
	**3-6 years**	28.02%
	**6-10 years**	11.50%
	**> 10 years**	1.30%
**Identified Risk factor(s)**	**Smoking**	38.46%
	**Dyslipidaemia**	32.06%
	**Hypertension**	29.07%
	**None**	22.27%
	**Obesity**	10.04%
**Comorbid condition(s) present**	**None**	64.86%
	**Chronic Kidney Disease**	25.51%
	**Peripheral Artery Disease**	9.80%
**Status on complication(s)**	**None**	47.13%
	**Nephropathy**	24.37%
	**Neuropathy**	23.24%
	**Myocardial Infarction**	16.68%
	**Stroke**	11.26%
	**Ischaemic Heart Disease**	5.59%
	**Retinopathy**	3.89%

BMI, body mass index; SD, standard deviation

### Efficacy analyses in overall population

**Fig 2:**
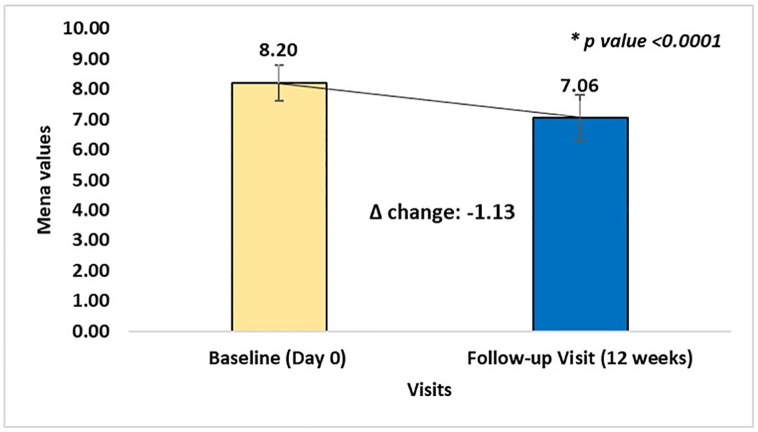
HbA1c improvement from baseline to 12 weeks of treatment. Mean change in HbA1c levels from baseline (Day 0) to 12-week follow-up in patients with type 2 diabetes mellitus (T2DM) receiving triple fixed-dose combination therapy. Bars represent mean values and error bars indicate standard deviation (SD). Mean value significantly decreased from 8.20 ± 0.60 at baseline to 7.06 ± 0.76 at follow-up showing a mean change (Δ) of −1.13 (*p* < 0.0001).

The triple-drug regimen significantly improved glycemic and metabolic parameters across the patient population. Mean HbA1c levels significantly dropped from 8.20 ± 0.60% at baseline to 7.06 ± 0.76% after 12 weeks of therapy (13.65% reduction, p < 0.0001) (Fig 2). The mean change in HbA1c was –1.13% [95% CI: –1.17 to –1.09]. Fasting blood glucose (FBG) levels declined from 188.02 ± 47.15 mg/dL at baseline to 168.54 ± 44.77 mg/dL at Week 4 and 146.01 ± 41.53 mg/dL at Week 12, corresponding to reductions of 10.44% and 21.67% respectively (P < 0.0001). The mean change in FBG at Week 12 was –42.01 mg/dL [95% CI: –43.89 to –40.13].

Similarly, 1-hour PPG decreased from 254.73 ± 53.01 mg/dL at baseline to 213.95 ± 49.92 mg/dL at 4 weeks (15.20% reduction, p < 0.0001) and 188.93 ± 42.90 mg/dL at 12 weeks (24.21% reduction, p < 0.0001) with mean change at Week 12 was –65.81 mg/dL [95% CI: –68.78 to –62.83], while 2-hour PPG dropped from 234.74 ± 50.40 mg/dL to 205.77 ± 48.22 mg/dL at 4 weeks (12.04% reduction, p < 0.0001) and 179.40 ± 42.51 mg/dL at 12 weeks (21.94% reduction, p < 0.0001) with a mean change of –55.34 mg/dL [95% CI: –58.04 to –52.64].

**Fig 3:**
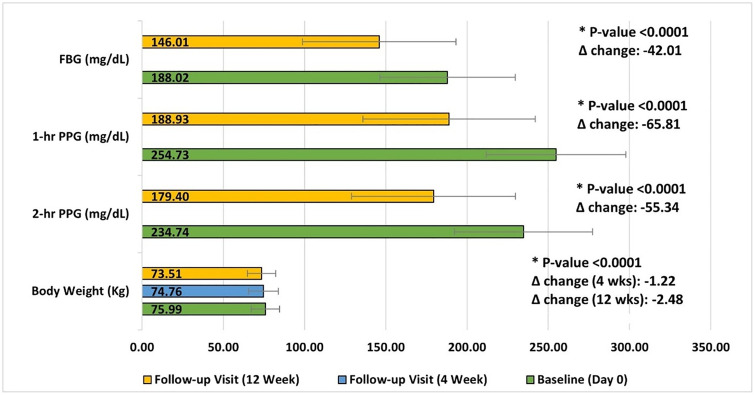
Improvement in baseline efficacy parameters post treatment with study drug. Mean Change in fasting blood glucose (FBG), 1-hour postprandial glucose (PPG), 2-hour PPG and body weight from baseline (Day 0) to follow-up visits at 4 weeks and 12 weeks. Bars represent mean values and error bars indicate standard deviation (SD). Significant reductions were observed across all parameters at 12 weeks compared with baseline (*p* < 0.0001). Mean changes (Δ) from baseline were −42.01 mg/dL for FBG, −65.81 mg/dL for 1-hour PPG, −55.34 mg/dL for 2-hour PPG and −2.48 kg for body weight.

Furthermore, body weight also showed a significant reduction from 75.99 ± 8.67 kg at baseline to 74.76 ± 9.07 kg at 4 weeks (1.60% reduction, p < 0.0001) and 73.51 ± 8.72 kg at 12 weeks (3.18% reduction, p < 0.0001). The mean change in weight was –1.22 kg [95% CI: –1.42 to –1.03] at Week 4 and –2.48 kg [95% CI: –2.70 to –2.26] at Week 12 (Fig 3). Additionally, serum albumin levels decreased significantly from 4.97 ± 1.63 g/dL at baseline to 4.58 ± 1.38 g/dL at 12 weeks (5.09% reduction, p < 0.0001) with mean change of –0.39 g/dL [95% CI: –0.44 to –0.35]. These findings highlight the regimen’s effectiveness in achieving better glycemic control and metabolic outcomes.

### Responder rates

**Fig 4:**
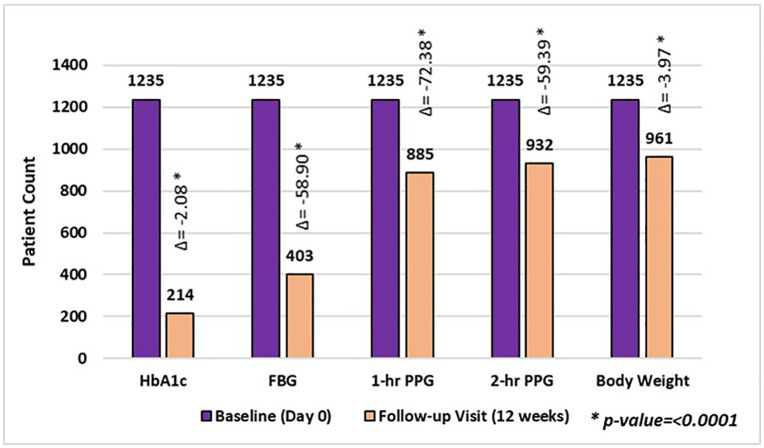
Responder’s rate in overall patients after 12 weeks of treatment. Responder rates for glycaemic parameters (HbA1c, fasting blood glucose [FBG], 1-hour postprandial glucose [PPG-1h], 2-hour postprandial glucose [PPG-2h]) and body weight from baseline to 12 weeks of follow-up. Bars represent the proportion of patients achieving clinically meaningful improvements in each parameter. Δ values denote the mean change from baseline. Statistically significant improvements were observed across all parameters (*p* < 0.0001).

A 12-week analysis demonstrated significant improvements in glycemic control across multiple parameters. Specifically, 17.33% of patients achieved HbA1c levels below 6.5%, with an average reduction of 2.08 (25.29% reduction; p < 0.0001). For FBG, 32.63% of patients reached target levels of less than 126 mg/dL, with a mean decrease of 58.90 mg/dL (33.32% reduction; p < 0.0001). In terms of PPG control, 71.66% of patients achieved the 1-hour target of less than 200 mg/dL, with a reduction of 72.38 mg/dL (27.81% reduction; p < 0.0001) and 75.47% of patients showed reduction in 2-hour PPG levels, with a decrease of 59.39 mg/dL (24.76% reduction; p < 0.0001). Additionally, 77.81% of participants experienced an average weight loss of 3.97 kg (5.18% reduction; p < 0.0001). (Fig 4)

### Multivariate multiple regression analysis

This study employed a multivariate multiple regression analysis to evaluate the collective impact of baseline clinical characteristics on multiple glycemic outcomes. Baseline HbA1c demonstrated a strong and statistically significant association with multivariate outcomes (Wilks’ Lambda = 0.875, p < 0.001) highlighting its importance in predicting glycemic improvement. Similarly, baseline fasting blood glucose (FBG) emerged as a very strong predictor (Wilks’ Lambda = 0.688, p<0.001), while postprandial glucose (PPG) values at 1 hour (Wilks’ Lambda = 0.543, p < 0.001) and 2 hours (Wilks’ Lambda = 0.528, p < 0.001) were identified as extremely strong predictors of glycemic response. Significant reductions in FBG and PPG not only contribute to lowering HbA1c but are also associated with reduced risk of diabetes-related comorbidities such as hypertension, dyslipidemia, chronic kidney disease and microvascular complications (retinopathy, neuropathy, nephropathy).

Baseline BMI (Wilks’ Lambda = 0.979, p < 0.001) and weight (Wilks’ Lambda = 0.987, p < 0.001) were also significant predictors, suggesting that anthropometric factors also contribute to treatment efficacy. Age (p = 0.382) and gender (p = 0.185) did not significantly affect outcomes, indicating that therapeutic response was largely independent of these demographic factors. (Table 2) Further details on multivariate multiple regression analysis are presented in S3 Table in S1 File.

**Table 2:** Multivariate regression analysis. Multivariate analysis of predictors influencing glycaemic and clinical outcomes. Results are presented as Wilks’ Lambda values with corresponding significance levels. Lower Wilks’ Lambda indicates stronger predictive effect on multivariate outcomes.

Predictor	Wilks’ Lambda	Sig.	Statistical interpretation
Baseline_HbA1c	0.875	< 0.001	Significant effect on multivariate outcomes
Age	0.997	0.382	Not significant
Baseline_BMI	0.979	< 0.001	Mild but significant effect
Gender	0.995	0.185	Not significant
Baseline_FBG	0.688	< 0.001	Very strong multivariate predictor
Baseline_Weight	0.987	< 0.001	Small but significant effect
Baseline_PPG_1hr	0.543	< 0.001	Extremely strong predictor
Baseline_PPG_2hr	0.528	< 0.001	Extremely strong predictor

### Subgroup analysis (BMI ≥ 23 kg/m² vs < 23 kg/m²)

In participants with a BMI ≥ 23 kg/m² (n = 1137), 17.68% achieved HbA1c levels below 6.5%, with an average reduction of 2.10% (25.41% decrease; 95% CI: –2.21 to –1.99; p < 0.0001). For FBG, 32.98% reached target levels of <126 mg/dL with a mean decrease of 59.65 mg/dL (33.52% reduction; 95% CI: –63.55 to –55.75; p < 0.0001). Additionally, 71.42% of participants achieved the 1-hour PPG target of <200 mg/dL, showing an average reduction of 72.90 mg/dL (27.94% reduction; 95% CI: –76.53 to –69.27; p < 0.0001), while 75.73% showed improvement in 2-hour PPG levels with a mean reduction of 59.68 mg/dL (24.85% reduction; 95% CI: –62.81 to –56.55 p < 0.0001). These findings indicate strong glycemic control in this group with statistically significant changes. For weight reduction, 78.72% of participants with a BMI ≥ 23 kg/m² achieved a 4.02 kg weight loss (5.17% reduction; 95% CI: –4.20 to –3.85; p < 0.0001),

In the BMI < 23 kg/m² group (n = 98), 67.35% of participants experienced a reduction in body weight of 3.32 kg (5.33% reduction; 95% CI: –3.79 to –2.84; p < 0.0001). For HbA1c, 13.27% reached the targeted level < 6.5%, with an average reduction of 1.78% (23.57% reduction; 95% CI: –2.16 to –1.41; p < 0.0001. For FBG, 28.57% attained target levels of <126 mg/dL, corresponding to a mean decrease of 48.86 mg/dL (30.63% reduction; 95% CI: –58.59 to–39.13; p < 0.0001). At 1-hour PPG, 74.49% of participants achieved the target of <200 mg/dL with an average reduction of 66.52 mg/dL (26.42% reduction; 95% CI: –76.53 to –56.51; p < 0.0001), while 72.45% showed improvement in 2-hour PPG, achieving the target of <200 mg/dL with a mean reduction of 55.93 mg/dL (23.68% reduction; 95% CI: –67.49 to –44.37; p < 0.0001) (Table 3).

**Table 3:** Changes in clinical and biochemical parameters from baseline to follow-up visits. Data are presented as mean paired differences with standard deviation, standard error of mean, 95% confidence intervals, and two-sided p-values. Parameters assessed include body weight, HbA1c, fasting blood glucose (FBG), postprandial glucose (PPG at 1h and 2h), serum creatinine, and serum albumin. Subgroup analyses were performed as a post hoc for patients with BMI < 23 and BMI ≥ 23. All p-values <0.0001 indicate statistically significant improvements compared to baseline.

Paired samples test							
Parameters	Visits	Paired differences			95% confidence interval of the difference		Two-Sided p
		Mean	Std. deviation	Std. error mean	Lower	Upper	
**Weight (kg)**	PB Week 4 - Baseline	-1.22	3.48	0.10	-1.42	-1.03	<0.0001
	PB Week 12 - Baseline	-2.48	3.94	0.11	-2.70	-2.26	<0.0001
**HbA1c (%)**	PB Week 12 - Baseline	-1.13	0.72	0.02	-1.17	-1.09	<0.0001
**FBG (mg/dL)**	PB Week 12 - Baseline	-42.01	33.70	0.96	-43.89	-40.13	<0.0001
**PPG (mg/dL)**	PB Week 12 - Baseline	-65.81	53.34	1.52	-68.78	-62.83	<0.0001
**PPG (mg/dL)**	PB Week 12 - Baseline	-55.34	48.40	1.38	-58.04	-52.64	<0.0001
**Sr. Creatinine (mg/dL)**	PB Week 12 - Baseline	-0.23	0.86	0.02	-0.27	-0.18	<0.0001
**Sr. Albumin (g/dL)**	PB Week 12 - Baseline	-0.39	0.82	0.02	-0.44	-0.35	<0.0001
**Sub-Group Analysis (BMI <23) (n=98)**							
**Weight (kg)**	PB Week 12 - Baseline	-3.32	1.94	0.24	-3.79	-2.84	<0.0001
**HbA1c (%)**	PB Week 12 - Baseline	-1.78	0.62	0.17	-2.16	-1.41	<0.0001
**FBG (mg/dL)**	PB Week 12 - Baseline	-48.86	25.09	4.74	-58.59	-39.13	<0.0001
**PPG (mg/dL)**	PB Week 12 - Baseline	-66.52	42.91	5.02	-76.53	-56.51	<0.0001
**PPG (mg/dL)**	PB Week 12 - Baseline	-55.93	48.86	5.80	-67.49	-44.37	<0.0001
**Sub-Group Analysis (BMI ≥23) (n=98)**							
**Weight (kg)**	PB Week 12 - Baseline	-4.02	2.66	0.09	-4.20	-3.85	<0.0001
**HbA1c (%)**	PB Week 12 - Baseline	-2.10	0.79	0.06	-2.21	-1.99	<0.0001
**FBG (mg/dL)**	PB Week 12 - Baseline	-59.65	38.37	1.98	-63.55	-55.75	<0.0001
**PPG (mg/dL)**	PB Week 12 - Baseline	-72.90	52.70	1.85	-76.53	-69.27	<0.0001
**PPG (mg/dL)**	PB Week 12 - Baseline	-59.68	46.77	1.59	-62.81	-56.55	<0.0001

### Patient satisfaction

Patient-reported outcomes revealed a high level of satisfaction with the treatment regimen with 96.92% of patients rating their experience as “good” or “very good” (62.43% and 34.49%, respectively). Additionally, treatment adherence was excellent with 90.04% of patients demonstrating adherence to the prescribed 12-week treatment regimen.

### Safety analysis

The treatment regimen demonstrated a favourable safety profile with 93.44% of patients experiencing no adverse events (AEs) throughout the study period. Notably, despite a high prevalence of comorbid cardiovascular conditions (50.67%, n = 636), no cardiovascular-related adverse events were reported indicating a good cardiovascular safety profile of the treatment regimen. A functional excel spreadsheet containing raw data of study is provided as S2 File.

## Discussion

This real-world, retrospective, observational chart review successfully met its pre-determined objectives, demonstrating improvements in HbA1c, FBG, 1- and 2-hour PPG levels and weight change. The study also evaluated drug utilization patterns, PGA for improvement, user experience and safety following treatment with the triple-drug combination tablets at 4 and 12 weeks as pre-specified. In line with ADA and RSSDI guidelines, triple therapy is particularly beneficial in managing postprandial glucose excursions, reducing HbA1c levels and providing durable glycaemic control. Overall, this strategy improves patient outcomes by offering a more robust and versatile treatment option [8,9]. For patients with insufficient glycaemic control on metformin and sulfonylurea (SU), adding a Dipeptidyl peptidase-4 (DPP-4) inhibitors as a third agent is a rational approach. DPP-4 inhibitors have a neutral to mild effect on body weight and hypoglycaemia risk, potentially offsetting the increased risks of weight gain and hypoglycaemia associated with SU [17]. Moreover, DPP-4 inhibitors have the potency to reduce HbA1c levels in Asian patients [18,19].

Comparative analysis of glycemic and metabolic outcomes across different therapeutic regimens highlights the superior efficacy of triple fixed-dose combination therapy. The START multicentric randomized controlled trial demonstrated modest improvements with dual therapy (glimepiride + metformin) over 12 weeks with mean reductions in HbA1c (0.42 ± 0.24%), FPG (12.41 ± 13.21 mg/dL), PPG (21.01 ± 21.88 mg/dL) and minimal weight change (0.15 ± 0.97 kg). Similarly, the sitagliptin + metformin group showed limited efficacy with mean reductions in HbA1c (0.30 ± 0.20%), FPG (7.45 ± 15.36 mg/dL), PPG (12.09 ± 28.22 mg/dL) and weight change (–0.22 ± 0.82 kg) [20]. In contrast, our study evaluating a triple fixed-dose combination of sitagliptin, glimepiride and metformin over the same duration demonstrated significantly greater improvements: HbA1c reduction of 1.13 ± 0.72%, FPG reduction of 42.01 ± 33.70 mg/dL, PPG reduction of 65.81 ± 53.34 mg/dL and weight reduction of 2.48 kg. These findings underscore the enhanced glycemic control and metabolic benefits of triple therapy, supporting its potential role in optimizing early treatment strategies for patients with T2DM. These findings are further supported by the TRIPLE-AXEL trial, which reported a mean HbA1c reduction of **–**2.03% at 24 weeks with triple therapy comprising metformin, a sodium-glucose cotransporter-2 (SGLT2) inhibitor and a dipeptidyl peptidase-4 (DPP-4) inhibitor. These evidence underscores the potential of triple oral therapy to deliver early, effective and clinically meaningful glycemic control in patients with T2DM [21].

In a placebo-controlled trial by Hermansen et al. [22], the efficacy of adding sitagliptin to glimepiride monotherapy or metformin–glimepiride combination therapy was evaluated over a 24-week period (N = 364 patients). The initial HbA1c level was 8.4%. After 24 weeks, the glimepiride–sitagliptin group showed a 0.3% reduction in HbA1c and 0.88 mg/dL in FBG. In contrast, the metformin–glimepiride–sitagliptin group achieved greater reductions: 0.59% in HbA1c, 7.8 mg/dL in FBG and 21.3 mg/dL in 2-hour PPG. Adding sitagliptin to combination therapy was more effective than glimepiride monotherapy. In another placebo-controlled trial by Ba J et al. [23] a subgroup analysis of 111 patients treated with metformin–SU and sitagliptin showed reductions of 0.86% in HbA1c, 22.2 mg/dL in FBG and 33.4 mg/dL in PPG after 24 weeks of treatment. These placebo-controlled trials demonstrated statistically significant reductions in glycemic parameters consistent with the results of the current study.

Downes MJ et al. [24] conducted a meta-analysis evaluating the efficacy of sitagliptin, metformin, and glimepiride in individuals with T2DM. Their findings indicated a reduction in HbA1c levels by 0.71%, compared to a greater reduction of 1.13% observed in the current study. Additionally, while the meta-analysis reported an increase in body weight by 0.71 kg, our study demonstrated a significant weight reduction of 2.48 kg. Similarly, Liu X et al [25] reported a mean HbA1c reduction of 0.70% with triple-drug therapy which was surpassed by our findings. Regarding 2-hour PPG, a study evaluating DPP-4 inhibitors as an early add-on therapy showed a reduction of 51.9 mg/dL after 24 weeks [26]. This aligns with our research which demonstrated a 2-hour PPG reduction of 55.34 mg/dL after 12 weeks of treatment. In a 52-week randomized, double-blind, active-controlled phase-3 study, participants on stable metformin and SU therapy were assigned to receive either canagliflozin or sitagliptin daily. The primary endpoint was the change in HbA1c from baseline at 52 weeks. Both canagliflozin and sitagliptin effectively reduced HbA1c levels. Comparative studies of combination therapies highlighted the positive efficacy of sitagliptin establishing it as a valuable treatment option in managing glycemic control compared to other OADs [27,28].

The triple combination therapy demonstrated notable benefits in BMI and weight management. Among patients with a BMI > 23 kg/m², 17.68% achieved an HbA1c level <7% (p < 0.0001), while those with a BMI < 23 kg/m² showed a 14.68% reduction in HbA1c (p < 0.0001). Despite the inclusion of glimepiride known for its association with weight gain, the therapy resulted in a significant mean weight loss of 3.18% from baseline (p < 0.0001). This aligns with evidence that sitagliptin when combined with metformin-SU combination mitigates the weight gain typically linked to SU [29]. In contrast, real-world data from the AWARE-2 study [30] demonstrated that GLP-1 receptor agonists combined with SGLT2 inhibitors provide superior improvements in glycemic control and weight reduction in obese, high-risk patients underscoring the comparative efficacy of newer injectable-based strategies. However, the present study results reinforce that oral triple fixed-dose combinations remain effective, low-cost alternatives, particularly in contexts where oral therapy is prioritized.

Improved glycemic control is a well-established factor in reducing cardiovascular risk among patients with type 2 diabetes mellitus (T2DM). In our study, the triple fixed-dose combination therapy led to substantial mean changes from baseline in glycemic parameters including HbA1c (–1.13 ± 0.72%), fasting blood glucose (–42.01 ± 33.70 mg/dL) and postprandial glucose (–55.34 ± 48.40 mg/dL). These improvements are clinically meaningful, as elevated postprandial and fasting glucose levels have been independently associated with increased risk of cardiovascular events including myocardial infarction and stroke. Recent evidence confirms that postprandial glucose is a stronger predictor of cardiovascular mortality. For instance, Hershon et al. (2019) and Cavalot et al. (2011) demonstrated that 2-hour postprandial glucose levels are significantly associated with cardiovascular events and all-cause mortality, even after adjusting for A1C and other risk factors [31,32]. Ceriello et al. emphasized that 2-hour postprandial glucose levels are linearly associated with cardiovascular mortality and interventions targeting PPG have demonstrated reductions in cardiovascular events in trials such as STOP-NIDDM and meta-analyses involving acarbose therapy [33]. These findings reinforce the importance of targeting both fasting and postprandial glycemia to reduce cardiovascular comorbidities in T2DM patients. Evidence from large-scale trials such as UKPDS and ADVANCE has shown that even modest reductions in HbA1c significantly lower the incidence of microvascular complications [34,35].

Angiotensin receptor blockers (ARBs) play a vital role in the management of diabetes, particularly in patients with nephropathy or at high risk of kidney disease, as they inhibit the renin–angiotensin–aldosterone system (RAAS) and reduce albuminuria, thereby providing renal protection [36]. In the present study, telmisartan was the most commonly prescribed agent. Telmisartan offers reno-protection by reducing albuminuria in CKD patients with diabetes [37]. Higher serum albumin levels have been associated with an increased risk of major adverse cardiovascular events (MACE). In a study by Zheng YY et al [38]. involving 14,994 coronary artery disease (CAD) patients serum albumin levels >5 g/dL were linked to a higher risk of MACE, while levels around 4.5 g/dL were associated with the lowest risk. Similarly, in the present study serum albumin levels decreased from 4.97 ± 1.63 g/dL at baseline to a normal range of 4.58 ± 1.38 g/dL after 12 weeks indicating potential cardiovascular benefits of the treatment. In the present study, CKD was the most prevalent comorbidity observed. A study by Cheng T et al [39]. demonstrated that serum albumin levels below 4.1 g/dL are strongly linked to poor renal outcomes and declining renal function. From a therapeutic perspective, preventing a decrease in albumin levels is a practical approach to slowing the progression of CKD.

T2DM is closely linked to accelerated atherosclerosis through mechanisms such as chronic inflammation, endothelial dysfunction and dyslipidemia, cardiovascular risk. Emerging evidence suggests that pharmacological interventions may also induce regression of atherosclerotic plaque. Bucciarelli et al. (2025) demonstrated that targeted therapies including lipid-lowering and anti-inflammatory agents can reduce coronary plaque burden, as assessed by coronary computed tomography angiography (CCTA). These findings underscore the importance of safe and effective therapeutic strategies in T2DM that offer both biochemical improvements and structural cardiovascular benefits through plaque modulation [40].

The triple fixed-dose combination therapy demonstrated a favorable short-term safety profile, with 93.44% of patients reporting no adverse events. Mild events such as URTI and nasopharyngitis were most common and serious adverse events were minimal (1.70%). Hypoglycaemia was reported in 0.48% of patients lower than expected for a sulfonylurea-containing regimen [41]. No cardiovascular adverse events were observed during the 12-week follow-up. In the context of integrated cardiometabolic care, recent tools such as the AWARE web application have facilitated rapid cardiovascular risk stratification in patients with T2DM, emphasizing the importance of integrating glycaemic control with proactive cardiovascular risk management [42].

Despite the wide range of antidiabetic medications, patient choice is essential in selecting treatment for chronic conditions like T2DM, as it requires balancing effectiveness with potential side effects [43]. FDCs that combine two or more drugs can improve treatment adherence and minimize AEs [44]. DPP-4 inhibitors provide significant glucose-lowering effects compared to a placebo in patients on SU. They also offer a favourable tolerability profile as part of oral triple therapy when added to a metformin-SU combination [45]. DPP-4 inhibitors are less effective than GLP-1 receptor agonists for reducing HbA1c and body weight [46]. Their oral route of administration and overall safety profile make them a practical option in routine clinical practice [47]. The robust cost-effectiveness data on FDCs in T2DM remain limited, their potential to reduce pill burden and simplify regimens may contribute to both clinical and economic advantages in routine care [48].

This study has several limitations. Its multicentre, retrospective, observational chart-review design may introduce patients’ selection bias as the individual with only completed and available medical records data were collected. Variability in laboratory and anthropometric measurements across study centres could influenced the outcomes. The absence of continuous glucose monitoring (CGM) restricted the ability to assess real-time glycemic fluctuations, whereas newer approaches such as intermittent interstitial glucose monitoring [49] and advanced hybrid closed loop systems [50] can provide more insights into glycemic variability. Additionally, the findings represent short-term outcomes (12 weeks) and may not be generalisable to long-term glycaemic, cardiovascular, or other comorbidity-related outcomes and safety profiles. Adverse events were recorded based on documented in medical records, which may be led to underreporting of such events such as hypoglycemia. Future research should incorporate prospective designs with longer follow-up durations, standardized data collection technique and CGM-based monitoring to enable a more comprehensive evaluation of glycemic control and safety. Emerging evidence on altered immune-metabolic responses in diabetes [51] highlights the need to explore these aspects in future studies to achieve a more holistic understanding of the therapeutic potential of this regimen in T2DM.

## Conclusion

The findings of this study indicate that the triple-drug combination of sitagliptin, glimepiride, and metformin is both effective and safe for managing glycemic control, reducing body weight and improving patient satisfaction in individuals with T2DM. Additionally, sitagliptin demonstrated significant efficacy in lowering FBG and HbA1c levels when combined with metformin and SU. This triple-drug combination offers a robust and multifaceted approach to managing T2DM, making it an ideal choice for normal or overweight patients requiring intensive glycemic control. Its proven efficacy and safety profile support its use as a preferred treatment option in clinical practice.

## Supporting information

S1 File S1, S2 and S3 Table. S1 Table contains Prescription analysis of prescribed dose of drug at baseline 4 week and 12 weeks of follow-ups and co-prescribed drugs for the comorbid condition. S2 Table contains Time of administration of fix dose combination (study drug) in patients with T2DM. S3 Table contains Multivariate regression analysis showing predictors of change in glycaemic parameters. Values represent regression coefficients (B), standard error, t-value, significance (p) and 95% confidence interval. Gender coded as 1 = female, 2 = male; parameter “0a” denotes reference category. *p* < 0.05 considered significant.
(DOCX)

S2 File S2 File contains raw data of GLIMSI Study. (XLSX)

## References

[1] Chawla M, Panneerselvam D, Gundgurthy A, Sud S, Alamchandani R, Aneja P, et al. Retrospective observational study on assessing sitagliptin and dapagliflozin as a fixed-dose combination in the indian population with type 2 diabetes mellitus: the SIDAXA study. Cureus. 2024;16(5):e60815. doi: 10.7759/cureus.60815 38910691 PMC11191412

[2] Shamanna P, Jha PK, Makwana A, Shukla H, Bavishi C. Observational, Multicenter, Retrospective, Study on the Usage Patterns of the Fixed Dose Combination of Glimepiride, Metformin, and Voglibose in Type 2 Diabetes Management. Cureus. 16: e52064. doi:10.7759/cureus.520643. 10.7759/cureus.52064 PMC10859676 38348001

[3] Kumar A, Gangwar R, Zargar AA, Kumar R, Sharma A. Prevalence of diabetes in India: a review of IDF diabetes Atlas 10th edition. Curr Diabetes Rev. 2024;20(1):e130423215752. doi: 10.2174/1573399819666230413094200 37069712

[4] DeFronzo RA, Ferrannini E, Groop L, Henry RR, Herman WH, Holst JJ, et al. Type 2 diabetes mellitus. Nat Rev Dis Primers. 2015;1:15019. doi: 10.1038/nrdp.2015.19 27189025

[5] Shillah WB, Yahaya JJ, Morgan ED, Bintabara D. Predictors of microvascular complications in patients with type 2 diabetes mellitus at regional referral hospitals in the central zone, Tanzania: a cross-sectional study. Sci Rep. 2024;14(1):5035. doi: 10.1038/s41598-024-55556-x 38424145 PMC10904798

[6] Galicia-Garcia U, Benito-Vicente A, Jebari S, Larrea-Sebal A, Siddiqi H, Uribe KB, et al. Pathophysiology of Type 2 diabetes mellitus. Int J Mol Sci. 2020;21(17):6275. doi: 10.3390/ijms21176275 32872570 PMC7503727

[7] Defronzo RA. Banting lecture. From the triumvirate to the ominous octet: a new paradigm for the treatment of type 2 diabetes mellitus. Diabetes. 2009;58(4):773–95. doi: 10.2337/db09-9028 19336687 PMC2661582

[8] Fisher R. American diabetes association releases 2023 standards of care in diabetes to guide prevention, diagnosis, and treatment for people living with diabetes. 2022.

[9] RSSDI clinical practice recommendations for the management of type 2 diabetes mellitus 2022. Int J Diabetes Dev Ctries. 2022;42(S1):1–143. doi: 10.1007/s13410-022-01129-5 PMC5838201 29527102

[10] Kim JY, Kim NH. Initial combination therapy in type 2 diabetes. Endocrinol Metab (Seoul). 2024;39(1):23–32. doi: 10.3803/EnM.2023.1816 38031401 PMC10901659

[11] Davies MJ, Aroda VR, Collins BS, Gabbay RA, Green J, Maruthur NM, et al. Management of hyperglycemia in type 2 diabetes, 2022. a consensus report by the American Diabetes Association (ADA) and the European Association for the Study of Diabetes (EASD). Diabetes Care. 2022;45(11):2753–86. doi: 10.2337/dci22-0034 36148880 PMC10008140

[12] Kalra S, Singh AK, Das S, Pendurthi B, Dharmadhikari S, Ahire P, et al. Sitagliptin as an add-on therapy to other glucose-lowering agents in patients with type 2 diabetes mellitus: a narrative review. J Assoc Physicians India. 2025;73(4):e13–8. doi: 10.59556/japi.73.0923 40200618

[13] Parmar N, Gupta AK, Jhaveri K, A B, Chhaya G, Kansara S, et al. Real-world assessment of personalized approach with voglibose fixed-dose combination in type 2 diabetes mellitus. Cureus. 2024;16(4):e57494. doi: 10.7759/cureus.57494 38707131 PMC11066517

[14] Hameed MF, Majeed SH. Comparison of sitagliptin, glimepiride, and metformin group with glimepiride and metformin group in treatment of diabetes mellitus type 2 patients. J Pharmacol Drug Dev. 2022;1:6–10.

[15] Vijay N, Amitabh S, Subrata C, Suhas E. Clinical experience with triple drug combination efficacy and patient treatment survey. Indian J Clin Pract. 2025.

[16] Manjula S, Krishna Kumar M. Expert opinion on fixed-dose combinations of Sitagliptin + metformin and the triple drug combination of Voglibose + Glimepiride + Metformin in the management of type 2 diabetes mellitus in Indian settings. Int J Endocrinol Sci. 2024;6(1):01–6. doi: 10.33545/26649284.2024.v6.i1a.6

[17] Ko SH, Hur KY, Rhee SY, Kim NH, Moon MK, Park SO, et al. Antihyperglycemic agent therapy for adult patients with type 2 diabetes mellitus 2017: a position statement of the Korean diabetes association. Diabetes Metab J. 2017;41(5):337–48. doi: 10.4093/dmj.2017.41.5.337 29086531 PMC5663672

[18] Park H, Park C, Kim Y, Rascati KL. Efficacy and safety of dipeptidyl peptidase-4 inhibitors in type 2 diabetes: meta-analysis. Ann Pharmacother. 2012;46(11):1453–69. doi: 10.1345/aph.1R041 23136353

[19] Kim YG, Hahn S, Oh TJ, Kwak SH, Park KS, Cho YM. Differences in the glucose-lowering efficacy of dipeptidyl peptidase-4 inhibitors between Asians and non-Asians: a systematic review and meta-analysis. Diabetologia. 2013;56(4):696–708. doi: 10.1007/s00125-012-2827-3 23344728

[20] Devarajan TV, Venkataraman S, Kandasamy N, Oomman A, Boorugu HK, Karuppiah SKP, et al. Comparative evaluation of safety and efficacy of glimepiride and sitagliptin in combination with metformin in patients with type 2 diabetes mellitus: indian multicentric randomized trial - START study. Indian J Endocrinol Metab. 2017;21(5):745–50. doi: 10.4103/ijem.IJEM_176_17 28989886 PMC5628548

[21] Kim NH, Moon JS, Lee Y-H, Cho HC, Kwak SH, Lim S, et al. Efficacy and tolerability of initial triple combination therapy with metformin, dapagliflozin and saxagliptin compared with stepwise add-on therapy in drug-naïve patients with type 2 diabetes (TRIPLE-AXEL study): a multicentre, randomized, 104-week, open-label, active-controlled trial. Diabetes Obes Metab. 2024;26(9):3642–52. doi: 10.1111/dom.15705 38853720

[22] Hermansen K, Kipnes M, Luo E, Fanurik D, Khatami H, Stein P, et al. Efficacy and safety of the dipeptidyl peptidase-4 inhibitor, sitagliptin, in patients with type 2 diabetes mellitus inadequately controlled on glimepiride alone or on glimepiride and metformin. Diabetes Obes Metab. 2007;9(5):733–45. doi: 10.1111/j.1463-1326.2007.00744.x 17593236

[23] Ba J, Han P, Yuan G, Mo Z, Pan C, Wu F, et al. Randomized trial assessing the safety and efficacy of sitagliptin in Chinese patients with type 2 diabetes mellitus inadequately controlled on sulfonylurea alone or combined with metformin. Journal of Diabetes. 2016;9(7):667–76. doi: 10.1111/1753-0407.12456 27502307

[24] Downes MJ, Bettington EK, Gunton JE, Turkstra E. Triple therapy in type 2 diabetes; a systematic review and network meta-analysis. PeerJ. 2015;3:e1461. doi: 10.7717/peerj.1461 26664803 PMC4675096

[25] Liu X, Wang L, Xing Y, Engel SS, Zeng L, Yao B, et al. Efficacy and safety of metformin and sitagliptin‐based dual and triple therapy in elderly Chinese patients with type 2 diabetes: subgroup analysis of STRATEGY study. J of Diabetes Invest. 2020;11: 1532–41.doi: 10.1111/jdi.13277 PMC7610099 32304283

[26] Goldstein BJ, Feinglos MN, Lunceford JK, Johnson J, Williams-Herman DE. Effect of initial combination therapy with sitagliptin, a dipeptidyl peptidase-4 inhibitor, and metformin on glycemic control in patients with type 2 diabetes. Diabetes Care. 2007;30(8):1979–87. doi: 10.2337/dc07-0627 17485570

[27] Khaloo P, Asadi Komeleh S, Alemi H, Mansournia MA, Mohammadi A, Yadegar A, et al. Sitagliptin vs. pioglitazone as add-on treatments in patients with uncontrolled type 2 diabetes on the maximal dose of metformin plus sulfonylurea. J Endocrinol Invest. 2018;42(7):851–7. doi: 10.1007/s40618-018-0991-0 30535871

[28] Schernthaner G, Gross JL, Rosenstock J, Guarisco M, Fu M, Yee J, et al. Canagliflozin compared with sitagliptin for patients with type 2 diabetes who do not have adequate glycemic control with metformin plus sulfonylurea: a 52-week randomized trial. Diabetes Care. 2013;36(9):2508–15. doi: 10.2337/dc12-2491 23564919 PMC3747923

[29] Sharma M, Beckley N, Nazareth I, Petersen I. Effectiveness of sitagliptin compared to sulfonylureas for type 2 diabetes mellitus inadequately controlled on metformin: a systematic review and meta-analysis. BMJ Open. 2017;7(10):e017260. doi: 10.1136/bmjopen-2017-017260 29084794 PMC5665259

[30] Berra C, Manfrini R, Bifari F, Cipponeri E, Ghelardi R, Centofanti L, et al. Improved glycemic and weight control with Dulaglutide addition in SGLT2 inhibitor treated obese type 2 diabetic patients at high cardiovascular risk in a real-world setting. The AWARE-2 study. Pharmacol Res. 2024;210:107517. doi: 10.1016/j.phrs.2024.107517 39613122

[31] Hershon KS, Hirsch BR, Odugbesan O. Importance of postprandial glucose in relation to A1C and cardiovascular disease. Clin Diabetes. 2019;37(3):250–9. doi: 10.2337/cd18-0040 31371856 PMC6640888

[32] Cavalot F, Pagliarino A, Valle M, Di Martino L, Bonomo K, Massucco P, et al. Postprandial blood glucose predicts cardiovascular events and all-cause mortality in type 2 diabetes in a 14-year follow-up: lessons from the San Luigi Gonzaga diabetes study. Diabetes Care. 2011;34(10):2237–43. doi: 10.2337/dc10-2414 21949221 PMC3177732

[33] Ceriello A. Postprandial hyperglycemia and cardiovascular disease: is the HEART2D study the answer?. Diabetes Care. 2009;32(3):521–2. doi: 10.2337/dc08-2209 19246590 PMC2646040

[34] Stratton IM. Association of glycaemia with macrovascular and microvascular complications of type 2 diabetes (UKPDS 35): prospective observational study. BMJ. 2000;321(7258):405–12. doi: 10.1136/bmj.321.7258.405 10938048 PMC27454

[35] Heller SR, ADVANCE Collaborative Group. A summary of the ADVANCE Trial. Diabetes Care. 2009;32 Suppl 2(Suppl 2):S357-61. doi: 10.2337/dc09-S339 19875581 PMC2811451

[36] Izzo JL, Zion AS. Value of Angiotensin receptor blocker therapy in diabetes. J Clin Hypertens (Greenwich). 2011;13(4):290–5. doi: 10.1111/j.1751-7176.2011.00447.x 21466628 PMC8673251

[37] Wang K, Hu J, Luo T, Wang Y, Yang S, Qing H, et al. Effects of angiotensin-converting enzyme inhibitors and angiotensin II receptor blockers on all-cause mortality and renal outcomes in patients with diabetes and albuminuria: a systematic review and meta-analysis. Kidney Blood Press Res. 2018;43(3):768–79. doi: 10.1159/000489913 29794446

[38] Zheng Y-Y, Wu T-T, Hou X-G, Yang Y, Yang H-T, Pan Y, et al. The higher the serum albumin, the better? Findings from the PRACTICE study. Eur J Intern Med. 2023;116:162–7. doi: 10.1016/j.ejim.2023.07.023 37532654

[39] Cheng T, Wang X, Han Y, Hao J, Hu H, Hao L. The level of serum albumin is associated with renal prognosis and renal function decline in patients with chronic kidney disease. BMC Nephrol. 2023;24(1):57. doi: 10.1186/s12882-023-03110-8 36922779 PMC10018824

[40] Bucciarelli L, Andreini D, Stefanini G, Fiorina RM, Francone M, Catapano F, et al. Pharmacological regression of atherosclerotic plaque in patients with type 2 diabetes. Pharmacol Res. 2025;213:107635. doi: 10.1016/j.phrs.2025.107635 39921019

[41] Sahay RK, Mittal V, Gopal GR, Kota S, Goyal G, Abhyankar M, et al. Glimepiride and metformin combinations in diabetes comorbidities and complications: real-world evidence. Cureus. 2020;12(9):e10700. doi: 10.7759/cureus.10700 33133865 PMC7594657

[42] Berra C, Manfrini R, Mirani M, Bucciarelli L, Zakaria AS, Piccini S, et al. AWARE A novel web application to rapidly assess cardiovascular risk in type 2 diabetes mellitus. Acta Diabetol. 2023;60(9):1257–66. doi: 10.1007/s00592-023-02115-x 37270748 PMC10359387

[43] Shields BM, Dennis JM, Angwin CD, Warren F, Henley WE, Farmer AJ, et al. Patient stratification for determining optimal second-line and third-line therapy for type 2 diabetes: the TriMaster study. Nat Med. 2023;29(2):376–83. doi: 10.1038/s41591-022-02120-7 36477733 PMC7614216

[44] Melikian C, White TJ, Vanderplas A, Dezii CM, Chang E. Adherence to oral antidiabetic therapy in a managed care organization: a comparison of monotherapy, combination therapy, and fixed-dose combination therapy. Clin Ther. 2002;24(3):460–7. doi: 10.1016/s0149-2918(02)85047-0 11952029

[45] Makrilakis K. The role of DPP-4 inhibitors in the treatment algorithm of type 2 diabetes mellitus: when to select, what to expect. IJERPH. 2019;16(15):2720. doi: 10.3390/ijerph16152720 31366085 PMC6696077

[46] Tran S, Retnakaran R, Zinman B, Kramer CK. Efficacy of glucagon‐like peptide‐1 receptor agonists compared to dipeptidyl peptidase‐4 inhibitors for the management of type 2 diabetes: a meta‐analysis of randomized clinical trials. Diabetes Obesity Metabolism. 2018;20(S1):68–76. doi: 10.1111/dom.13137 29364587

[47] Scheen AJ. DPP-4 inhibitors in the management of type 2 diabetes: a critical review of head-to-head trials. Diabetes Metab. 2012;38(2):89–101. doi: 10.1016/j.diabet.2011.11.001 22197148

[48] Lokhandwala T, Smith N, Sternhufvud C, Sörstadius E, Lee WC, Mukherjee J. A retrospective study of persistence, adherence, and health economic outcomes of fixed-dose combination vs. loose-dose combination of oral anti-diabetes drugs. J Med Econ. 2016;19(3):203–12. doi: 10.3111/13696998.2015.1109518 26473990

[49] Rossi A, Rossi G, Montefusco L, Cimino V, Pastore I, Gandolfi A, et al. A new glucose monitoring system for the intermittent monitoring of interstitial glucose values in patients with diabetes mellitus. J Diabetes Metab Disord. 2024;23(2):2201–5. doi: 10.1007/s40200-024-01488-2 39610481 PMC11599681

[50] Rossi A, Montefusco L, Reseghetti E, Pastore IF, Rossi G, Usuelli V, et al. Daytime hypoglycemic episodes during the use of an advanced hybrid closed loop system. Diabetes Res Clin Pract. 2023;206:111011. doi: 10.1016/j.diabres.2023.111011 37956944

[51] D’Addio F, Lazzaroni E, Lunati ME, Preziosi G, Ercolanoni M, Turola G, et al. Vaccinome landscape in nearly 620 000 patients with diabetes. J Clin Endocrinol Metab. 2025;110(5):e1590–7. doi: 10.1210/clinem/dgae476 39040010 PMC12012803

