# Supplementary material for: GLIMSI: A real-world, multicenter study assessing the effectiveness and safety of Sitagliptin + Glimepiride + Metformin FDC in Indian patients with Type 2 diabetes
Source: PLoS One. 2026 Feb 6;21(2):e0337107. doi: 10.1371/journal.pone.0337107 (PMC12880711; doi:10.1371/journal.pone.0337107)
Supplement: S1 File — S2 Table contains Time of administration of fix dose combination (study drug) in patients with T2DM. S3 Table contains Multivariate regression analysis showing predictors of change in glycaemic parameters. Values represent regression coefficients (B), standard error, t-value, significance (p) and 95% confidence interval. Gender coded as 1 = female, 2 = male; parameter “0a” denotes reference category. p < 0.05 considered significant. (DOCX) [file pone.0337107.s001.docx]

**S1 Table**

| **Variable** | | | | **N=1235** |
| --- | --- | --- | --- | --- |
| **Drug and Dose** | | **Baseline** | **Week 4** | **Week 12** |
| **Sulfonylurea (mg)** | **0.5** | 0.81% | 7.69% | 4.21% |
|  | **1** | 50.45% | 52.15% | 59.27% |
|  | **2** | 33.93% | 31.66% | 30.28% |
|  | **3** | 7.69% | 4.78% | 4.78% |
|  | **4** | 7.13% | 3.72% | 1.46% |
| **Metformin (mg)** | **500** | 6.72% | 14.57% | 27.37% |
|  | **1000** | 89.64% | 80.24% | 69.72% |
|  | **1500** | 3.64% | 1.70% | 1.21% |
|  | **2000** | 0.00% | 3.48% | 1.70% |
| **Sitagliptin (mg)** | **50** | 79.84% | 80.57% | 86.23% |
|  | **100** | 20.16% | 19.43% | 13.77% |
| **Co-prescribed agents for comorbid condition(s)** | | | | |
| **ARBs** | | **None** | | 29.80% |
|  |  | **Telmisartan** | | 64.13% |
|  |  | **Losartan** | | 6.07% |
| **Statins** | | **None** | | 56.76% |
|  |  | **Rosuvastatin** | | 23.08% |
|  |  | **Atorvastatin** | | 20.16% |
| **Anticoagulants** | | **None** | | 75.95% |
|  |  | **Aspirin** | | 14.09% |
|  |  | **Clopidogrel** | | 9.39% |
|  |  | **Prasugrel** | | 0.97% |
|  |  | **Ticagrelor** | | 0.81% |
| **ARB, angiotensin II receptor blocker; NA, not available** | | | | |

**S2 Table**

| **Time of Administration** | |
| --- | --- |
| **Only Morning** | 60.32% |
| **Only Afternoon** | 17.00% |
| **Only Night** | 11.82% |
| **Morning and Night** | 10.85% |

**S3 Table**

| **Multivariate Regression Analysis** | | | | | | | |
| --- | --- | --- | --- | --- | --- | --- | --- |
| **Dependent Variable** | **Covariates or Parameter** | **B** | **Std. Error** | **t** | **Sig.** | **95% Confidence Interval** | |
|  |  |  |  |  |  | **Lower Bound** | **Upper Bound** |
| **HBA1C_Change** | Intercept | 2.34 | 0.34 | 6.80 | <0.001 | 1.66 | 3.01 |
|  | Baseline_Hba1C | -0.42 | 0.03 | -12.96 | <0.001 | -0.49 | -0.36 |
|  | Age | 0.00 | 0.00 | -0.10 | 0.918 | 0.00 | 0.00 |
|  | Baseline_BMI | -0.02 | 0.01 | -2.93 | 0.004 | -0.03 | -0.01 |
|  | Baseline_Weight | 0.01 | 0.00 | 2.81 | 0.005 | 0.00 | 0.02 |
|  | Baseline_FBG | 0.00 | 0.00 | 1.99 | 0.047 | 0.00 | 0.00 |
|  | Baselien_PPG_1Hrs | 0.00 | 0.00 | -4.05 | <0.001 | 0.00 | 0.00 |
|  | Baseline_PPG_2hrs | 0.00 | 0.00 | 1.88 | 0.06 | 0.00 | 0.00 |
|  | [Gender=1.00] | 0.04 | 0.05 | 0.83 | 0.406 | -0.05 | 0.13 |
|  | [Gender=2.00] | 0a | . | . | . | . | . |
| **FBG_Change** | Intercept | 72.33 | 14.69 | 4.93 | <0.001 | 43.52 | 101.14 |
|  | Baseline_Hba1C | -5.84 | 1.39 | -4.21 | <0.001 | -8.57 | -3.12 |
|  | Age | 0.06 | 0.08 | 0.70 | 0.482 | -0.10 | 0.22 |
|  | Baseline_BMI | 0.29 | 0.30 | 0.96 | 0.338 | -0.30 | 0.88 |
|  | Baseline_Weight | -0.06 | 0.14 | -0.39 | 0.693 | -0.33 | 0.22 |
|  | Baseline_FBG | -0.34 | 0.02 | -17.67 | <0.001 | -0.38 | -0.30 |
|  | Baselien_PPG_1Hrs | -0.07 | 0.02 | -3.23 | 0.001 | -0.12 | -0.03 |
|  | Baseline_PPG_2hrs | 0.04 | 0.02 | 1.88 | 0.06 | 0.00 | 0.08 |
|  | [Gender=1.00] | -3.50 | 1.99 | -1.76 | 0.079 | -7.41 | 0.41 |
|  | [Gender=2.00] | 0a | . | . | . | . | . |
| **PPG_1HRs_Change** | Intercept | 126.47 | 20.00 | 6.32 | <0.001 | 87.24 | 165.70 |
|  | Baseline_Hba1C | -6.17 | 1.89 | -3.26 | 0.001 | -9.88 | -2.46 |
|  | Age | 0.07 | 0.11 | 0.62 | 0.539 | -0.15 | 0.29 |
|  | Baseline_BMI | 1.16 | 0.41 | 2.82 | 0.005 | 0.35 | 1.96 |
|  | Baseline_Weight | -0.22 | 0.19 | -1.13 | 0.261 | -0.59 | 0.16 |
|  | Baseline_FBG | 0.00 | 0.03 | -0.10 | 0.918 | -0.05 | 0.05 |
|  | Baselien_PPG_1Hrs | -0.76 | 0.03 | -24.87 | <0.001 | -0.82 | -0.70 |
|  | Baseline_PPG_2hrs | 0.14 | 0.03 | 4.53 | <0.001 | 0.08 | 0.19 |
|  | [Gender=1.00] | -0.51 | 2.71 | -0.19 | 0.851 | -5.83 | 4.81 |
|  | [Gender=2.00] | 0a | . | . | . | . | . |
| **PPG_2Hrs_Change** | Intercept | 88.70 | 19.23 | 4.61 | <0.001 | 50.98 | 126.43 |
|  | Baseline_Hba1C | -2.83 | 1.82 | -1.55 | 0.121 | -6.39 | 0.74 |
|  | Age | -0.10 | 0.11 | -0.96 | 0.339 | -0.31 | 0.11 |
|  | Baseline_BMI | -0.02 | 0.39 | -0.06 | 0.956 | -0.79 | 0.75 |
|  | Baseline_Weight | 0.19 | 0.19 | 1.01 | 0.314 | -0.18 | 0.55 |
|  | Baseline_FBG | 0.09 | 0.03 | 3.38 | <0.001 | 0.04 | 0.14 |
|  | Baselien_PPG_1Hrs | 0.02 | 0.03 | 0.75 | 0.456 | -0.04 | 0.08 |
|  | Baseline_PPG_2hrs | -0.64 | 0.03 | -22.26 | <0.001 | -0.70 | -0.58 |
|  | [Gender=1.00] | -0.39 | 2.61 | -0.15 | 0.882 | -5.50 | 4.73 |
|  | [Gender=2.00] | 0a | . | . | . | . | . |
| **“a” This parameter is set to zero because it is redundant. [Gender 1: Female, 2: Male]** | | | | | | | |
